# Mitochondrial signatures of infant mesenchymal stem cells predict child adiposity: The Healthy Start Study

**DOI:** 10.21203/rs.3.rs-9476945/v1

**Published:** 2026-05-11

**Authors:** Lauren E. Gyllenhammer, Madeline Rose Keleher, Cheyret Wood, Ivana V. Yang, Jacob E. Friedman, Thomas Jansson, Dana Dabelea, Katerina Kechris, Kristen E. Boyle

**Affiliations:** University of California, Irvine; University of Colorado Anschutz; University of Colorado Anschutz; University of Colorado Anschutz; University of Oklahoma Health Sciences; University of Colorado Anschutz Medical Campus; The Lifecourse Epidemiology of Adiposity and Diabetes (LEAD) Center; University of Colorado Anschutz; University of Colorado Anschutz

## Abstract

**Importance:**

Although obesity risk is multifactorial, identification of molecular pathways at birth may reveal early-life susceptibility, guiding prevention and intervention efforts.

**Objective:**

We measured transcriptional and DNA methylation profiles of umbilical cord-derived mesenchymal stem cells (MSCs), which are progenitors for body composition (*e.g.*, adipose, muscle), and tested associations with childhood adiposity over the first 4-6yr of life.

**Design:**

Among 140 mother/child dyads enrolled in The Healthy Start Cohort Study, MSCs were isolated at birth and analyzed for their transcriptomic (RNAseq) and DNA methylation profile (Illumina EPIC). We measured newborn (24-72hrs after birth, n=134), infant (4-6mo, n=128), and early childhood (4-6yr, n=81) adiposity (%fat mass [%FM]) with air displacement plethysmography. A parallel *in vitro* adiposity phenotype was modeled as triglyceride accumulation during MSC adipogenesis.

**Setting:**

Prenatal obstetrics clinics at the University of Colorado Hospital in 2010–2014. Follow-up of women and children is ongoing.

**Participants:**

Singleton infants born to healthy women across the BMI spectrum.

**Exposure:**

Newborn MSC transcriptome.

**Main Outcome:**

Infant/childhood adiposity (%FM).

**Results:**

302 MSC transcripts were associated with %FM at birth, infancy and early childhood (*p*=5, *p*=296 and *p*=1), respectively [false discovery rate, FDR<0.05]). Geneset Enrichment Analysis of transcriptome data revealed 670 pathways associated with %FM (FDR<0.05, *p*=3 at birth, *p*=267 infancy, *p*=400 early childhood). Gene sets involved in extracellular matrix organization, were positively associated, while mitochondrial fatty acid beta-oxidation, the citric acid (TCA) cycle, cell cycle and chromosome/telomere maintenance were negatively associated with adiposity at 4-6mo and 4-6yr (FDR<0.05). Mitochondrial complex I pathways downregulation was associated with higher *in vivo* adiposity at all three timepoints (FDR<0.05), and with *in vitro* adiposity (p<0.05). We examined shared core enrichment genes between infant and early childhood pathways (*p=*664), as input for targeted methylation analysis. Of these shared genes, DNA methylation in four CpGs (FDR<0.2) and one noteworthy gene region, *HDAC4* (FDR=0.055), associated with %FM in infancy and early childhood, respectively.

**Conclusions and Relevance:**

Newborn MSC transcriptomic and methylation features, particularly within mitochondrial pathways, were associated with adiposity through early childhood. These findings suggest that early-life mitochondrial signatures may predict biological susceptibility to greater fat mass accretion.

## INTRODUCTION

Over recent decades, overweight and obesity have risen sharply across all racial, ethnic and socioeconomic groups, impacting 1 in 5 children and 2 in 5 adults.^1,2^ This is largely attributed to dramatic shifts in environmental exposures and health behaviors, often referred to as an “obesogenic environment.”^3^ Despite near-universal exposure to these conditions, individuals vary considerably in their susceptibility to obesity and related co-morbidities, likely shaped by complex interplay of genetic, developmental, and behavioral factors.^4–6^ Understanding inter-individual vulnerability is critical, as it offers a window into targeted prevention and personalized treatment approaches.

Many studies have investigated the molecular mechanisms of obesity risk heterogeneity. However, most human studies examined pathways in the context of established obesity, limiting causal inference.^7^ Cells and tissues collected at birth are particularly useful for investigating molecular mechanisms contributing to the development of obesity before the onset of excess adiposity. Prior studies relied on placental or cord-blood cells,^6,8–18^ which provide important but indirect insight into metabolic tissue development and function. In contrast, our prior findings,^19–23^ along with others,^24,25^ have established human infant umbilical cord–derived mesenchymal stem cells (MSCs) as a model for investigating multiple metabolic precursors underlying obesity risk (reviewed here^26^). MSCs are progenitors for mesodermal tissues, including adipose and skeletal muscle, and thus are particularly relevant for investigating metabolic pathways underlying obesity susceptibility. Previously, we focused on hypothesis-driven MSC features, demonstrating adipogenic drivers such as zinc finger protein (Zfp)423 and peroxisome proliferator-activated receptor (PPAR)γ are influenced by fetal exposures (e.g., maternal obesity).^19,27^ Furthermore, baseline differences in MSC lipid accumulation and handling prospectively predicted adiposity through early childhood.^21,22^ These sub-phenotyping approaches offer potential for screening predefined pathways underlying obesity, but remain challenging to investigate *in vivo* prior to obesity development.

In the present study, we expand our cohort and apply an unbiased, transcriptome-wide approach to test the hypothesis that MSC transcriptomic and epigenomic features present at birth associate with longitudinal child adiposity. We tested prospective association of the newborn MSC transcriptome in 140 children from birth through 4–6 years of age. Transcriptome results then informed a targeted DNA methylation analysis. To complement *in vivo* adiposity measures, we additionally leveraged an *in vitro* MSC adiposity phenotype, lipid accumulation during adipogenesis, as proof of concept that MSC transcriptomic signatures align with intrinsic fat accretion. With this parallel framework and an unbiased transcriptomic approach, we identified known and novel pathways and metabolic signatures present at birth that may underlie susceptibility to later adiposity.

## METHODS

### Population

We collected MSCs from a convenience sample of 165 infants born to mothers participating in the longitudinal Healthy Start Study (Clinical Trials.gov, NCT02273297)^19^ as part of the ancillary Healthy Start BabyBUMP Project, as described previously.^28^ Briefly, eligible participants were ≥ 16 years old, pregnant with a singleton carry, and ≤ 23 weeks gestation. Exclusions included prior diabetes, preterm birth, or serious psychiatric illness. The Colorado Multiple Institutional Review Board approved the study, and all participants provided informed consent. The described procedures were conducted in accordance with the Declaration of Helsinki.

**Figure 1.**
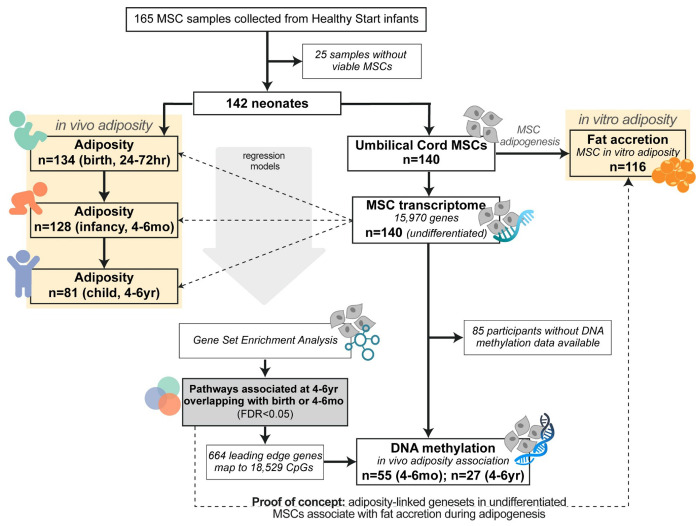
Study Workflow. This diagram summarizes the multi-stage dataset used to examine how the transcriptional and epigenetic features of umbilical cord–derived mesenchymal stem cells (MSC) related to early-life adiposity. MSCs were collected from 165 Healthy Start participants, yielding 140 viable undifferentiated MSC transcriptomes (15,970 genes). Adiposity measures were available for 134 newborns (birth, 24–72 hours), 128 infants (4–6 months), and 81 children (4–6 years). DNA methylation (CpG) data were available for a subset of participants (n=55 at 4–6 months; n=27 at 4–6 years). Gene set enrichment analyses identified pathways associated with adiposity that overlapped across time points (false discovery rate <0.05), with 664 leading-edge genes mapping to 18,529 CpG sites. A proof-of-concept adipogenesis model (n=116 MSC samples) was used to test whether adiposity-linked gene sets in undifferentiated MSCs also related to fat accretion during in-vitro differentiation. Together, the workflow demonstrates convergence between *in-vivo* adiposity measures and *in-vitro* MSC adipogenesis.

Of the 165 children with MSCs collected at birth, 142 had MSCs that were viable for culture to collect transcriptomic data. Of these participants, 140 have matching adiposity data available for at least 1 timepoint and are included in the current report: birth (n = 134), infancy (4-6mo; n = 128) and early childhood (4–6 year; n = 82). Of the 82 participants with available data in early childhood, one was excluded for non-biologically plausible adiposity (< 1% fat mass), resulting in a sample size of 81 participants. Of these children, MSC methylation data was available in a smaller subset (n = 55 at 4-6mo, and n = 27 at 4–6 year), and *in vitro* MSC adiposity was available in n = 116. Figure 1 shows the study workflow.

### Maternal and offspring phenotyping and body composition measurement

Healthy Start Study maternal phenotyping has been published elsewhere.^28^ Offspring birth weight, sex, and gestational age at birth were obtained from medical records. Child weight, length, age at scan and body composition (adiposity = percent fat mass [%FM], percent fat-free mass [%FFM]; whole-body air plethysmography [PEA POD and BOD POD; COSMED, Inc.]) were measured at each postnatal visit (birth [24-72hrs after birth], infancy [4-6mo], and early childhood [4-6yr]).

### Mesenchymal stem cell collection

The MSC culture and isolation procedures have been previously described.^19^ We cultured MSCs from fresh umbilical cord tissue explants, tested for purity based on established markers^29^ and conducted analyses on cells within passages 3–5.

### RNA sequencing and DNA methylation

Undifferentiated MSC pellets were flash frozen and stored at −80C; DNA and RNA were isolated using the Qiagen AllPrep kit. We performed RNA-seq in two batches, both using Nugen mRNA kit for library preparation, the first on Illumina HiSeq4000 with 1x150bp reads and the second with 2x150bp flow cell runs on Illumina NovaSEQ 6000, with 40 million reads/sample with batch correction. We measured DNA methylation via bisulfite conversion on the Illumina EPICv1 Array. Details available in **Supplementary Methods**.

#### In vitro MSC adiposity phenotype

We modeled *in vitro* MSC adiposity as previously described,^21^ via adipocyte triglyceride (TG) accumulation after 21 days of adipogenesis (referred to as MSC-TG).

### Statistical analyses

We conducted statistical analyses using R version 4.1.3. For outcomes with non-normal distributions, we applied a log_2_ transformation. Due to inconsistencies in Y-linked gene expression, offspring sex was determined from RNA-Seq expression (see Supplement).

### Untargeted transcriptomic analyses

We quantified gene counts using Ensembl annotation for GrCh 38 (version 86, accessed 08/23/2017), filtering those with < 10 average reads/sample, leaving 15,970 genes. We tested association of these genes and %FM with gene expression as the outcome, in order to account for the distribution of RNA-Seq counts using a negative binomial model, adjusting for offspring sex and age at measurement. For newborn scans, we used gestational age. For models where infant sex or age was significantly associated with FM% (P < 0.05), we performed sensitivity analyses testing association of adjusted %FM (%FM minus random effect age or sex) with transcript data to verify these covariates were no longer significant. We applied the Benjamini-Hochberg false discovery rate (FDR) correction.^30^

### Gene Set Enrichment Analysis

We performed gene set enrichment analysis (GSEA) for Gene Ontology (GO) and Reactome pathways using Fast Gene Set Enrichment Analysis using fgsea for R,^31^ with transcripts ranked by log_10_ p-value and direction of association. Because GSEA includes all transcripts, it enables detection of coordinated shifts across transcripts within a pathway while retaining directionality. We identified leading edge genes (core genes driving pathway enrichment, see Supplement) in significant pathways (FDR < 0.05) and evaluated overlap of 4–6 year associations with at least one other timepoint using a Fisher’s exact test. These overlapping genes were used as input for the subsequent targeted methylation analyses (p = 664 genes).

### Targeted methylation analyses

We performed data processing and quality control as detailed previously.^32^ 18,529 probes mapped to the 664 leading edge genes identified by GSEA. This targeted probe list was used to examine MSC DNA methylation associations with child %FM at 4-6mo and at 4–6 year, with linear models adjusted for child sex and age. We applied FDR correction^30^ to identify differentially methylated probes (DMPs). To test for differentially methylated regions (DMRs), we used Methylated CpGs Set Enrichment Analysis in R (mCSEA ver. 1.18.0).

### Post hoc pathway analysis with in vitro MSC adiposity phenotype

To support functional interpretation, we performed post hoc analyses using leading edge genes from the six mitochondrial GO pathways that consistently showed associations with %FM. We calculated summed z-scores of leading-edge genes counts for each pathway and correlated these results with the *in vitro* MSC adiposity phenotype (MSC-TG).

## RESULTS

Maternal and child characteristics are presented in Table 1 and do not differ notably from all potentially eligible participants in Healthy Start (**Supplemental Table 1**). Race and ethnicity frequencies were similar to the U.S. childbearing population (NCHS 2019),^33^ supporting the generalizability of our findings. About half of the women entered pregnancy at normal weight (n = 71, 51%), and the birth %FM is similar to previous reports;^34^ however, the %FM at 4-6mo and 4–6 year was lower.^35^

### MSC transcripts are associated with adiposity at each postnatal assessment

**Figure 2.**
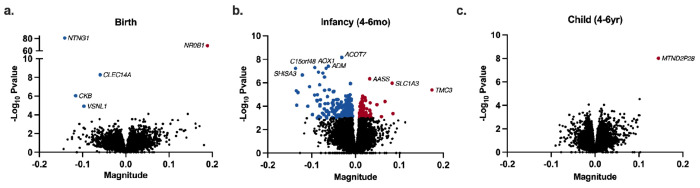
MSC transcriptomic analysis of genes associated with longitudinal child adiposity The associations between newborn mesenchymal stem cell (MSC) mRNA transcripts with percent fat mass (%FM) at birth (a) infancy (4-6 mo) (b) and early childhood (4-6 yr) (c). Significant transcripts with positive association with %FM are highlighted red, significant negative associations are highlighted blue (FDR<0.05). Magnitude represents the expected change in gene expression for 10% increase in fat mass.

We tested associations of mRNA transcripts with %FM at each postnatal assessment. 302 MSC transcripts were associated with childhood adiposity from birth through 4–6 years of age (Fig. 2, **Supplemental Tables S2a, S3–S5**; FDR < 0.05). Specifically, there were 5 transcripts associated with %FM at birth, 296 associated at 4-6mo, and 1 gene MTND2P28, positively associated at 4–6 year (Fig. 2, Table 2).

#### GSEA findings show mitochondrial genes consistently associate with adiposity through 4–6 years

**Figure 3.**
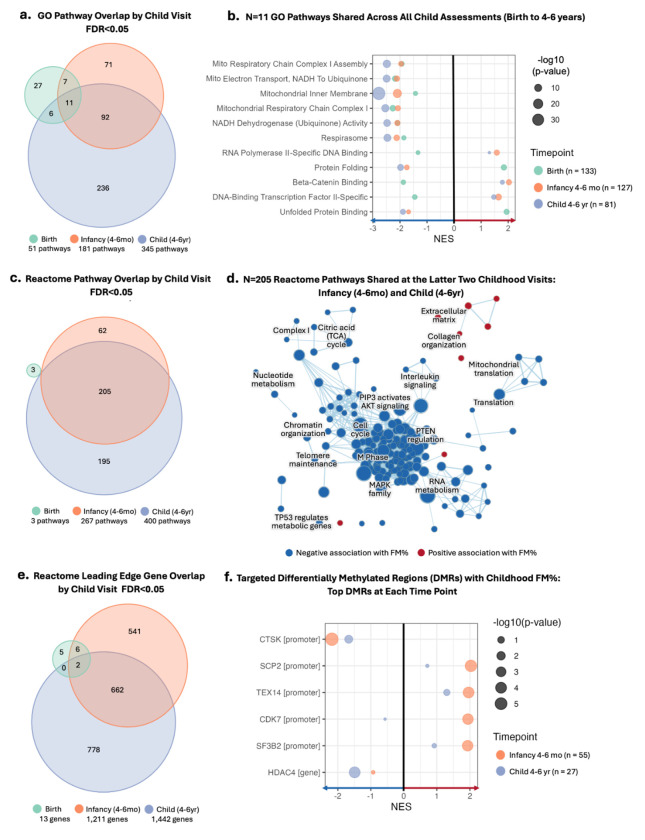
MSC GSEA pathways and DNA methylation associate with adiposity through 4-6 years We conducted gene set enrichment analysis (GSEA) for transcript association with percent fat mass (%FM) at the three postnatal assessments and interrogated overlapping pathways using Gene Ontology (GO) (a) and Reactome databases (c) FDR<0.05). There were 11 GO processes that significantly related to %FM at all three timepoints (b), and 205 Reactome pathways shared between %FM at infancy (4-6mo) and early childhood (4-6yr) (d; FDR<0.05). Next, we examined the overlap of leading-edge genes shared between significant infant and early childhood pathways (e) as input for targeted methylation analysis, and examined the overlap of the top differentially methylated DNA regions (DMR) with %FM at infancy (4-6mo) and early childhood (4-6yr) (f; all have FDR<0.05 for at least one timepoint, except *HDAC4* FDR=0.055). *NES= normalized enrichment score, which reflects the strength and direction of coordinated enrichment of predefined gene or genomic feature sets across the ranked transcript or probe list.

We conducted GSEA for transcript association with %FM at the three postnatal assessments using Reactome and GO databases. A total of 670 Reactome, and 577 GO processes associated with %FM from birth to 4–6 year (FDR < 0.05; in **Supplemental Tables S2b, S7–S8**). Of these, the largest number of pathways associated with %FM occurred at the 4–6 year timepoint (Reactome = 400, GO = 345, Fig. 3a, 3c).

Next, we searched for pathways that showed consistent relationships with %FM from birth through 4–6 year (i.e., across all three child assessments). There were 11 significant GO pathways, with 6 of these processes related to downregulation in mitochondrial function (e.g., mitochondrial respiratory chain complex I (CI), respirasome) (FDR < 0.05, Fig. 3b, **Supplemental Table S9)**. Additionally, there were 2 Reactome pathways that associated with %FM across all three assessments, both of which related to downregulated mitochondrial function (i.e., fatty acid beta-oxidation, CI biogenesis), though the association was not statistically significant (FDR < 0.2, **Supplemental Table S9**).

In addition, there were a large number of shared pathways associated with %FM 4-6mo and 4–6 year assessments (FDR < 0.05; Reactome N = 205, GO = 103; Fig. 3a, 3c, **Supplemental Table S10**). The enrichment analyses for the shared Reactome pathways are illustrated in Fig. 3d; we observed that these pathways showed the same direction of effect across time points. Overall, we found upregulation of pathways involved in extracellular matrix (ECM) and collagen synthesis (e.g., ECM proteoglycans, collagen biosynthesis) and downregulation of pathways related to the mitochondria (e.g., citric acid cycle, CI biogenesis, respiratory electron transport), cell cycle, DNA stability and telomere function (e.g., chromosome and telomere maintenance, TP53 DNA damage response), and metabolic signaling (e.g., AKT signaling, PTEN Regulation, MAPK family). In addition, there were several altered pathways related to stem cell differentiation and fate (e.g., beta-catenin, WNT, RUNX2 signaling). These Reactome pathways are complemented by GO pathway analyses showing similar shared downregulation in mitochondrial oxidative phosphorylation, upregulated ECM and collagen organization and altered signaling pathways involved in pluripotent stem cell fate and regulation (**Supplemental Table S10**).

#### Targeted Methylation Analyses

Next, we examined the leading-edge genes shared between significant infant and early childhood pathways, as input for targeted methylation analysis. From the untargeted Reactome GSEA (FDR < 0.05) there were 1,211 leading edge genes for the 4-6mo infant time point, and 1,442 leading edge genes from the 4–6 year early childhood timepoint. 664 genes were on both lists (Fig. 3e, **Supplemental Table S11**); 18,529 probes mapped to these genes for the subsequent targeted methylation analysis. Of these shared genes, DNA methylation in four CpGs, linked to *NDUFB2, GNS, EXOSC10* and *TOMM7*, nominally associated with %FM in infancy (FDR < 0.2, **Supplemental Tables S2c, S12-13**). In addition, 14 DMRs nominally associated with %FM in infancy and early childhood (FDR < 0.1), including one noteworthy gene region, *HDAC4*, (FDR = 0.055) (Fig. 3f, **Supplemental Tables S2c, S14-15**). Of these DMRs, 5 promoters were significantly associated with %FM in infancy, *CTSK, SCP2, CDK7, TEX14*, and *SF3B2* (FDR < 0.05), and of these, *CTSK* demonstrated paired nominal associations with %FM in early childhood (p < 0.05). Notably the methylation changes uncovered here (with 1 exception) were common at both the 4-6mo and 4–6 year timepoints.

### Mitochondrial genes are inversely associated with in vitro MSC adiposity phenotype

**Figure 4.**
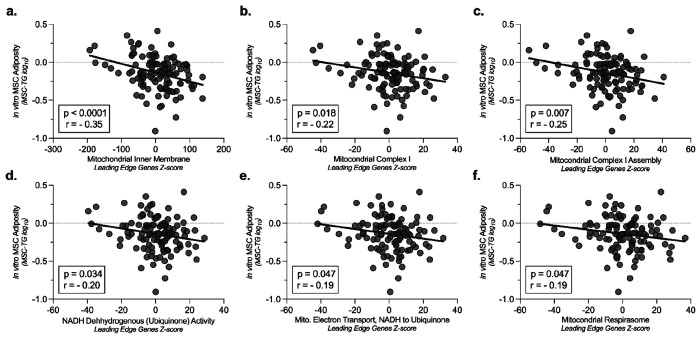
Mitochondrial genes are inversely associated with *in vitro* MSC adiposity phenotype Six mitochondrial GO pathways showing consistent directional associations with child adiposity were selected and the summed z-score of leading-edge genes was calculated for each. Correlations between MSC mitochondrial z-score and *in vitro* MSC adiposity phenotype (MSC-TG), measured as triglyceride accumulation after 21 days of adipogenic differentiation, are shown (a-f) (p<0.05).

Using the parallel *in vivo-in vitro* adiposity framework, we next tested whether pathways linked to child adiposity also mapped onto MSC intrinsic fat accretion upon adipogenesis. We selected six mitochondrial GO pathways, each showing consistent associations with child adiposity and matched directionally across all time points, for post hoc proof of concept functional analysis (see pathways in **Table 3a**). As shown in Fig. 4, the summed z-score of leading-edge genes from these pathways showed significant correlations with *in vitro* MSC adiposity (MSC-TG; p < 0.05, r = −0.19 to −0.33), reflecting the same directional pattern observed with *in vivo* adiposity.

## DISCUSSION

This study identified transcriptomic and epigenomic profiles in MSCs at birth that prospectively associated with adiposity through early childhood. We observed downregulation of mitochondrial pathway genes in MSCs from children with higher adiposity across all timepoints. Moreover, the parallel *in vivo-in vitro* adiposity framework showed these same mitochondrial genes associated with greater fat accumulation in the *in vitro* MSC adiposity model, highlighting a link between mitochondrial MSC signatures and intrinsic propensity for fat accretion. Targeted methylation analyses further revealed decreased methylation of *HDAC4*, a histone deacetylase that regulates mitochondrial biogenesis and function;^36^ notably, HDAC inhibitors are in development for the treatment of diabetes and its complications.^36^ Taken together, these findings further highlight the role of mitochondrial genes and DNA methylation patterns as early precursors of childhood adiposity.

We observed fewer individual transcripts associated with adiposity at 4–6 years than 4–6 months, consistent with reduced statistical power due to the smaller sample size at the later timepoint. We therefore performed GSEA, which leverages the full, ranked distribution of tested genes to detect coordinated, modest shifts across biologically related pathways even when individual transcript associations are more subtle.^37^ The shared pathways across timepoints supports biological consistency, underscoring that differences in individual transcript results likely reflect changes in power across time rather than underlying biology.

A key finding is the consistent inverse association of MSC mitochondrial pathways with adiposity, including respiratory chain CI, fatty acid β-oxidation, and the mitochondrial inner-membrane. These findings parallel an *in vivo* study of weight-discordant monozygotic twins reporting downregulation of mitochondrial transcriptional signatures of adipose tissue from heavier versus leaner siblings; most notably reporting downregulation in OXPHOS and fatty acid β-oxidation transcriptomic pathways and CI protein levels.^38^ We extend these findings by demonstrating prospective associations in these same mitochondrial pathways, measured before the onset of excessive adipose tissue accumulation or overt obesity, with subsequent childhood adiposity levels. This highlights their potential role as early predictors rather than mere correlates of established obesity. *In vitro* studies support a causal relationship. For example, impaired CI activity increases adipocyte fat accumulation, as CI inhibition drives dosedependent triglyceride accumulation in 3T3-L1 pre-adipocytes.^39^ Our *in vitro* MSC adiposity phenotype reinforces this, as undifferentiated MSCs with lower mitochondrial transcript levels accumulated more lipid during adipogenesis. Interestingly, the only individual gene associated with adiposity at 4–6 y was *MTND2P28*, a non-coding mitochondrial pseudogene with potential RNA-mediated regulatory mechanisms, such as methylation of both nuclear and mitochondrial DNA.^40,41^ How *MTND2P28* contributes to early adiposity remains unknown and merits future study.

We also observed upregulation of ECM and collagen synthesis transcripts in association with offspring adiposity. ECM remodeling is essential for adipose tissue expansion, and ECM accumulation and fibrosis have been implicated in obesity-related metabolic dysfunction.^42^ These processes may be primed in MSCs at birth, potentially influencing adipose tissue development and function in early life. Consistent with this, we identified hypomethylation of cathepsin K (*CTSK*) associated with greater adiposity at both childhood timepoints. *CTSK*, a cysteine protease involved in ECM remodeling, is upregulated in adipose tissue of adults with obesity^43^ and promotes adipocyte differentiation.^44^ Both genetic deletion^45^ and pharmacologic inhibition of CTSK^46,47^ reduce adiposity and improve glucose metabolism in animal models, highlighting *CTSK* as a potential therapeutic target for obesity and related metabolic disorders.

Targeted methylation analysis revealed a link between DNA methylation of *HDAC4* and child adiposity. Prior studies reported differences in *HDAC4* methylation in children with obesity^48,49^ and altered expression in adipose tissue and blood cells of adults with obesity.^50^ Our data are the first to demonstrate a prospective relationship between *HDAC4* methylation at birth. *HDAC4* regulates inflammatory and metabolic processes by deacetylating transcription factors,^51^ and plays a role in mitochondrial biogenesis and function.^36^ Several epigenome-wide association studies (EWAS) have examined newborn DNA methylation in relation to birthweight or adiposity.^6,11–18^ Most used cord blood and weight-based adiposity proxies (e.g., BMI percentile), while a few used cord tissue containing MSCs^6^ or direct adiposity measures beyond birth (e.g., DEXA).^13,15,18^ None have assessed transcriptomics or isolated MSCs. Across multiple studies, ^6,14,15,17^ including our own,^18^ pathways related to cell cycle regulation, stem cell fate signaling, mitochondrial metabolism, and ECM remodeling are consistently associated with child adiposity. For example, Alfano et al.^14^ reported methylation differences in *ARID5B* and *KLF9*, transcriptional regulators of chromatin accessibility and adipocyte differentiation, and in *AURKC*, a key cell cycle regulator. These findings functionally overlap with our MSC transcriptomic pathways involving transcriptional and differentiation regulators (*RUNX*, *WNT*/β-catenin, *NOTCH*, Hedgehog, *TP53)*, and cell cycle components (*AURKA* and *PLK1)*. Methylation differences in *ARID5B* also appeared in two other EWAS studies,^11,12^ while in our study MSC *ARID5B* expression showed a trend-level relationship with adiposity across all child time points (p < 0.1). Despite cell heterogeneity and limited recurrence of specific methylation sites (reviewed here^52–54^), these studies suggest that early-life molecular features, captured via methylation or transcriptomics, converge on shared pathways that may influence adiposity trajectories.

Limitations to our study include modest sample size at later timepoints, particularly for methylation data. Additionally, these analyses were performed in young children of predominantly European ancestry, with relatively low adiposity and obesity rates, limiting generalizability. Replication in older, more diverse children with broader adiposity and geographic variation is needed. Our study strengths include a well-characterized longitudinal cohort with precision adiposity measures, integration of transcriptomic and epigenomic data, and rigorous statistical design and analyses. Unlike most studies estimating adiposity by BMI, which poorly detects excess body fat in early childhood,^55,56^ we measured body composition using air displacement plethysmography, improving interpretability of our molecular associations.

This study is the first to interrogate unbiased transcriptomic profiles of newborn MSCs associated with repeated adiposity measures from birth through 6 years of age. By using purified progenitor cells of adipose and muscle lineages rather than mixed-cell samples, we captured molecular signatures present at birth that precede tissue development and obesity onset. This approach revealed mitochondrial and ECM-related pathways as early molecular determinants of adiposity, supported by functional evidence linking MSC transcriptomic profiles to *in vitro* fat accumulation. These findings position mitochondrial and ECM pathways as potential early biomarkers or therapeutic targets and underscore MSCs as a valuable model for studying developmental origins of obesity.

## Supplementary Material

This is a list of supplementary files associated with this preprint. Click to download.

• GyllenhammerMSCRNASeqSupplementaryMethodsMarch27.docx

• GyllenhammerMSCRNAseqandchildadipositySupplementalTablesMarch2026.xlsx

## Figures and Tables

**Table 1:** Maternal and child characteristics MSC= mesenchymal stem cell; data are mean ± standard deviation (SD), unless otherwise stated.

(*n = 140*)	Mean (SD) or n (%)
**Mother**	
Age	28.6 (6.1)
Primiparous, n	72 (51%)
Pre-pregnancy BMI (kg/m^2^)	25.0 (5.0)
Women with obesity, n	19 (14%)
Race and ethnicity, n	
Non-Hispanic White	80 (57%)
Hispanic	36 (26%)
Non-Hispanic Black	14 (10%)
Other	10 (7%)
**Child**	
Females, n	59 (42%)
Gestational age at birth (wks)	39.5 (1.2)
MSC time to confluence (d)	27.1 (7.8)
Birth weight (g)	3272 (429)
Fat mass (kg) at birth (*n = 134*)	0.31 (0.2)
Fat mass (%) at birth (*n = 134*)	9.6 (4.1)
Age at 4–6 month visit (mo)	4.8 (0.9)
Weight (kg) at infancy 4-6mo visit (*n = 129*)	6.8 (0.9)
Fat mass (kg) at infancy 4-6mo visit (*n = 128*)	1.6 (0.5)
Fat mass (%) at infancy 4-6mo visit (*n = 128*)	24.7 (5.6)
Age at child visit (yrs)	4.6 (0.2)
Weight (kg) at child 4–6 year visit (*n = 85*)	17.8 (3.7)
Fat mass (kg) at child 4–6 year visit (*n = 81*)	3.5 (2.1)
Fat mass (%) at child 4–6 year visit (*n = 81*)	19.4 (7.0)

**Table 2.** Top 5 MSC transcripts associated with child adiposity

Birth % Fat Mass				
Ensembl Gene ID	Gene Symbol	Magnitude^a^	p-value	FDR
ENSG00000162631	*NTNG1*	−1.42	7.60E-82	1.21E-77*
ENSG00000169297	*NR0B1*	1.89	4.40E-69	3.54E-65*
ENSG00000176435	*CLEC14A*	−0.59	5.20E-09	0.00003*
ENSG00000166165	*CKB*	−1.16	9.10E-07	0.00360*
ENSG00000163032	*VSNL1*	−0.97	1.20E-05	0.03900*
Infancy (4–6 mo) % Fat Mass				
Ensembl Gene ID	Gene Symbol	Magnitude^a^	p-value	FDR
ENSG00000097021	*ACOT7*	−0.31	6.90E-09	0.00011*
ENSG00000148926	*ADM*	−0.62	4.00E-08	0.00019*
ENSG00000166920	*C15orf48*	−0.93	5.10E-08	0.00019*
ENSG00000138356	*AOX1*	−0.67	5.70E-08	0.00019*
ENSG00000178343	*SHISA3*	−1.37	5.90E-08	0.00019*
Early Childhood (4–6 year) % Fat Mass				
Ensembl Gene ID	Gene Symbol	Magnitude^a^	p-value	FDR
ENSG00000225630	*MTND2P28*	1.44	9.70E-09	0.00015*
ENSG00000105664	*COMP*	1.01	2.90E-05	0.23000
ENSG00000173418	*NAA20*	−0.15	8.70E-05	0.37000
ENSG00000183853	*KIRREL1*	0.2	9.20E-05	0.37000
ENSG00000163257	*DCAF16*	0.14	2.00E-04	0.38000

a Magnitude= Expected change in gene expression for 10% increase in fat mass; FDR= Benjamini-Hochberg false discovery rate (FDR) correction;

* FDR < 0.05 significance level

## Data Availability

The data that support the findings of this study are available from the corresponding author, [KB], upon reasonable request.

## Key Points

### Question:

Do transcriptomic and epigenomic features from newborn mesenchymal stem cells (MSCs) prospectively associate with child adiposity?

### Findings:

Human umbilical cord-derived MSCs are progenitors for tissues determining body composition (*e.g*., adipose, muscle). In a longitudinal cohort study of 140 mother/child dyads, the transcription and methylation profiles of MSCs were longitudinally associated with childhood adiposity over the first 4-6yr of life. Pathway analysis demonstrated that downregulation of mitochondrial pathways is associated with elevated %FM from birth to early childhood.

### Meaning:

MSC mitochondrial transcriptomic and DNA methylation signatures present at birth are longitudinal predictors of fat mass accretion.
